# Expression of matrix metalloproteinase 2 (MMP-2), E-cadherin and Ki-67 in metastatic and non-metastatic canine mammary carcinomas

**DOI:** 10.1186/s13620-016-0068-3

**Published:** 2016-08-02

**Authors:** Marcin Nowak, Janusz A. Madej, Bartosz Pula, Piotr Dziegiel, Rafal Ciaputa

**Affiliations:** 1 Department of Pathology, Faculty of Veterinary Medicine, Wroclaw University of Environmental and Life Sciences, 50-375 Wroclaw, Poland; 2 Department of Histology and Embryology, Wroclaw Medical University, 50-368 Wroclaw, Poland; 3 Department of Physiotherapy, Wroclaw University School of Physical Education, 51-612 Wroclaw, Poland

**Keywords:** Matrix metalloproteinase-2, E-cadherin, Ki-67, Mammary carcinoma, Metastasis, Dog

## Abstract

**Background:**

The aim of the study was to demonstrate the immunohistochemical expression of proteins that affect the metastatic potential of a tumour, including matrix metalloproteinase 2 (MMP-2) and E-cadherin. Another objective was to determine their correlation with the expression of the Ki-67 antigen in metastasizing and non-metastasizing mammary carcinomas in female dogs. The study was conducted on 32 canine mammary carcinomas (12 metastatic and 20 non-metastatic), classified as simple tubular and tubulopapillary carcinomas. Immunohistochemistry was performed to evaluate the expression of MMP-2, E-cadherin and Ki-67 antigen.

**Results:**

MMP-2 was expressed in 85 % of the non-metastatic tumours and in all the metastatic tumours, while E-cadherin was expressed in 85 % of the non-metastatic tumours and in 66 % of the metastatic tumours. The Ki-67 antigen was expressed in 65 % of the non-metastatic tumours and in 91 % of the metastatic tumours. The mean Ki-67 expression was slightly higher in tumours that had metastasized (1.5 ± 0.90 vs 1.1 ± 0.94; *p* = 0.22). A similar relationship was found in terms of the intensity of the MMP-2 expression (2.9 ± 1.9 vs 2.7 ± 2.4; *p* = 0.50). A decrease in the expression of E-cadherin (2.8 ± 2.5) was found in metastatic tumours compared to the expression in non-metastatic tumours (3.2 ± 2.3). However, these differences were not statistically significant (*p* = 0.63).

**Conclusion:**

We did not show significant differences in MMP-2, E-cadherin and Ki-67 expression between metastatic and non-metastatic tumours due to low number of cases studied, however further experiments are necessary to assess the role of these antigens in the process of canine mammary tumours metastasis.

## Background

In order to invade, the neoplastic cell released from a primary tumour has to overcome a dense extracellular matrix (ECM) of proteins comprised of collagen, laminin, fibronectin, vitronectin and many other particles, which together form the basement membrane (BM). This membrane forms a mechanical barrier between the epithelium or endothelium and the surrounding tissue [1]. The ability of tumour cells to degrade components of the ECM is directly associated with their metastatic potential [2]. Matrix metalloproteinases (MMPs) are enzymes that play an important role in the destruction of the ECM. Metalloproteinases are Zn^2+^ dependent proteases [3], which are mainly produced in fibroblasts, monocytes, leukocytes, macrophages, neutrophils, endothelial cells and are secreted by tumour cells [4, 5]. The vascular endothelial growth factor (VEGF), tumour necrosis factor alpha (TNF-α), interleukin-1 (IL-1) and prostaglandin (PG) were shown to stimulate the synthesis of MMPs [5]. The gelatinases, particularly MMP-2 and MMP-9, are thought to play an important role in carcinogenesis. Their increased expression and correlation with unfavourable prognostic factors have been found in human and canine breast cancer [6–12]. Those proteinases lead to a breakdown of type IV collagen, which constitutes the basement membrane scaffolding, including that of the vascular endothelium. This facilitates the intravasation of tumour cells, which is an important stage of metastasis.

The mechanism through which a cell metastasizes and adheres to the target site is not fully understood. However, this process always involves the release of a tumour cell from the primary malignancy. Cells require various chemical signals (cytokines, chemokines, hormones, neurotransmitters) to begin migration. Cellular adhesion molecules (CAM), located in cell membranes, play an important role at this stage of the metastatic cascade. The level of their expression influences the strength of connections between the neighbouring cells [13, 14]. There are four main classes of adhesion molecules: cadherins, integrins, selectins and immunoglobulin-like particles. Cadherin plays an important role in the invasion and metastasis of the tumour [15]. It is a calcium-dependent transmembrane protein [8]. Three major molecules are included in this group: E-cadherin (Epithelial-cadherin), N-cadherin (Neuronal cadherin) and P-cadherin (Placental cadherin). Cadherins connect to neighbouring cells by binding to other cadherins through an extracellular N-terminal amino acid sequence of His-Ala-Val. The cytoplasmic domain of E-cadherin binds to a group of interconnected proteins, called catenins (α, β and γ). β and γ-catenins compete with one another and directly bind to E-cadherin. α-catenin, on the other hand, connects E-cadherin to actin F and α-actinin, which constitute the cell cytoskeleton [14]. Any functional disturbances in the cadherin-catenin complex (dysfunction and/or a lack of cadherin or catenin) reduce cell adhesion and disturb cellular differentiation. This leads to an increase in the invasive potential of the tumour and may support metastasis [16]. It has been shown that transfection of a murine mammary tumour cell line with E-cadherin cDNA decreases its invasiveness [17]. This underlines the importance of E-cadherin in the acquisition of a malignant phenotype of tumour cells.

The aim of this study was to demonstrate the expression (tumour cell positivity as well as expression intensity) of proteins associated with the metastatic potential of mammary gland carcinomas, i.e., MMP-2 and E-cadherin. We also aimed to determine the enhancement of their expression when correlated with the expression of the Ki-67 antigen in canine mammary carcinomas. The expression of Ki-67 can be observed already during G1 phase of the cell cycle; it increases markedly during S and G2 phases, reaches its peak level during M phase, and is absent in G0 cells. Consequently, Ki-67 is detected mostly in proliferating cells [18, 19]. Furthermore, the expression of the studied markers in metastatic tumours was compared to the results obtained from the tumours that had not metastasized.

## Methods

The material for the study was sampled during surgery from 32 female dogs of various breeds, 8 to 14 years old. The tumours were verified as carcinomas based on a histopathological examination according to the classification by Goldschmidt et al. [20] as simple tubular (*n* = 17) and simple tubulopapillary carcinomas (*n* = 15) (12 cases with lymph node metastasis – diagnosed based on a fine needle biopsy, and 20 cases without metastasis following a 2 year observation). In order to confirm or rule out metastasis, the female dogs were examined 2 months after the surgical tumour removal. Thoracic radiography and abdominal ultrasonography were carried out in each dog. In every case, samples from the superficial inguinal or/and axillary lymph nodes (which have not been removed during mastectomy) were obtained by using a fine needle biopsy. Those examinations were repeated every 6 months.

Formalin-fixed, paraffin-embedded tissues were freshly cut into 4-μm- thick sections and mounted on Superfrost Plus slides (Menzel Gläser, Braunschweig, Germany). The sections were then dewaxed with xylene and gradually hydrated in alcohol. The activity of the endogenous peroxidase was blocked by a 5 min exposure to 3 % H_2_O_2_. The sections were then boiled in a microwave oven for 15 min in an Antigen Retrieval Solution [21, 22] (DakoCytomation, Glostrup, Denmark). In order to measure the levels of the studied antigens, the following antibodies were applied for 1 hour at room temperature (RT) in the following concentrations: polyclonal rabbit anti-MMP-2 (1:100; Chemicon Millipore, Billerica, MA, USA), monoclonal mouse anti-E-cadherin antibody (clone NCH-38, 1:150; DakoCytomation), monoclonal mouse anti-Ki-67 (clone MIB-1, 1:100; DakoCytomation). All the utilized antibodies were diluted in the Background Reducing Antibody Diluent (DakoCytomation). Next, the samples were incubated (15 min, RT) with secondary biotinylated antibodies and a streptavidin-horseradish peroxidase complex (LSAB2, HRP, DakoCytomation). The 3,3'-diaminobenzidine (7 min, room temperature, DakoCytomation) served as a substrate for the reaction. All the sections were counterstained with Meyer’s haematoxylin. In all the cases, controls were assessed and the specific antibody was substituted with the Primary Negative Control (DakoCytomation). Microphotographs of all the studied tumours were subjected to a computer-aided image analysis *via* a computer coupled to a BX53 optical microscope (Olympus, Tokyo, Japan). The set was able to record images and to analyse them digitally. The measurements were carried out using the Cell^A^ software (Olympus Soft Imaging Solution GmbH, Germany).

The expression of MMP-2 and E-cadherin was appraised using the modified semiquantitative IRS scale according to Remmele and Stegner [12, 23]. The method takes into account both the proportion of positively stained tumour cells and the intensity of the reaction colour, while its final result represents the product of both parameters, with values ranging from 0 to 12 points (no reaction = 0 points (−); weak reaction = 1–2 points (+), moderate reaction = 3–4 points (++), intense reaction = 6–12 points (+++)). The expression of the Ki-67 antigen was evaluated quantitatively by estimating the percentage of positive tumour cells (0–5 % = no reaction (0 points), 6–25 % = weak reaction (1 point), 26–50 % = moderate reaction (2 points), above 50 % = intense reaction (3 points)). For purpose of the statistical analysis the final IRS scores of MMP-2 and E-cadherin, as well as Ki-67 score for particular cases were used for deriving the mean values. All the analyses were performed by two independent pathologists, and in case of discrepant results, the sections were re-evaluated until a consensus was achieved.

The results were subjected to a statistical analysis using Prism 5.0 (GraphPad, La Jolla, CA, USA). The differences between the two groups were compared using the non-parametric test of Mann-Whitney, whereas associations between the expression of the analysed markers were assessed using the Spearman correlation and Fisher exact test. The results were considered significant at *p* < 0.05 in all the analyses. For purpose of statistical analysis the expression of studied antigens was dichotomized into low (none, low and moderate expression of studied markers) and high (intense expression) expressing tumours.

## Results and discussion

**Fig. 1:**
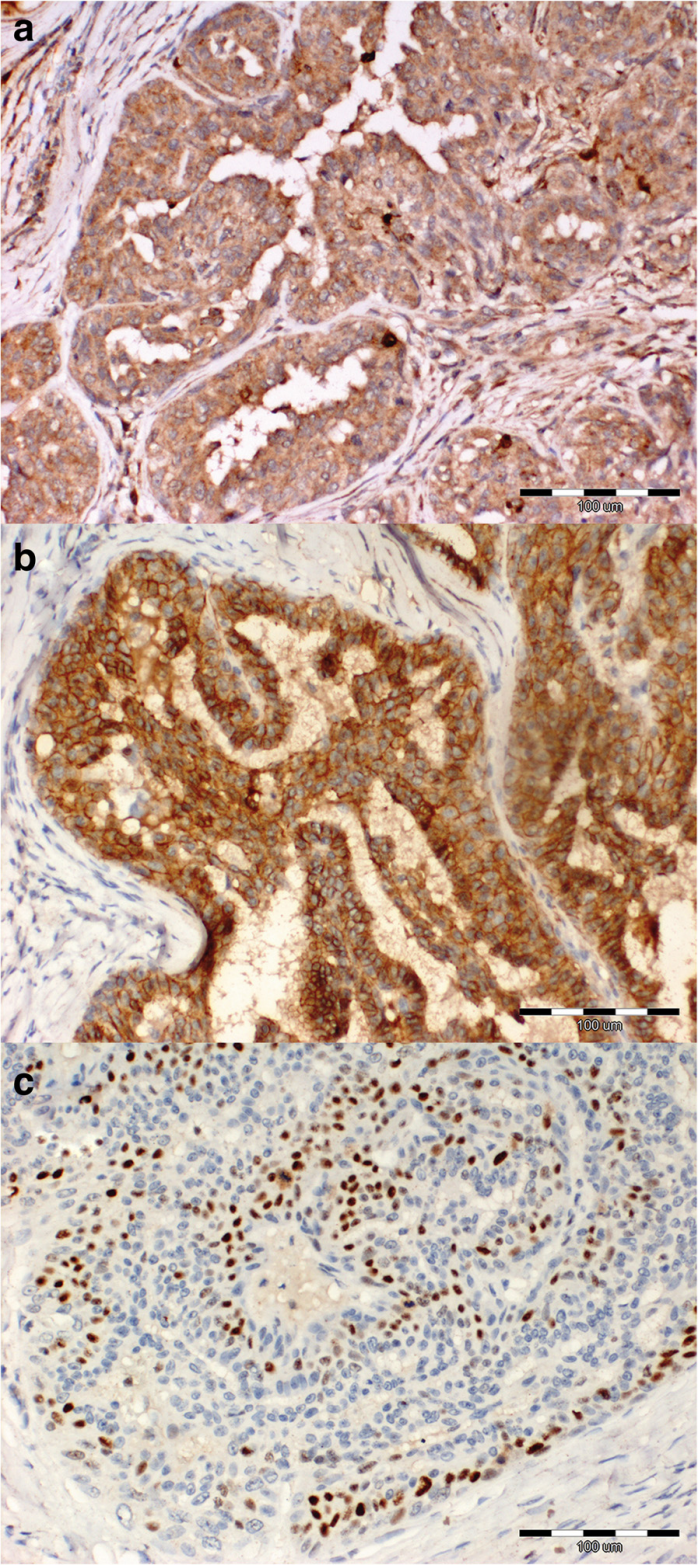
Immunoexpression of studied cell markers in canine mammary gland carcinoma. **a** High cytoplasmic expression of MMP-2 in carcinoma cells (simple tubular carcinoma, non-metastatic). **b** E-cadherin expression was predominantly found in the membrane of neoplastic cells (simple tubulopapillary carcinoma, non-metastatic). **c** Expression of Ki-67 localized in tumour cell nuclei (simple tubular carcinoma, metastatic)

MMP-2 was expressed in tumour cells in 17 out of 20 (85 %) of the non-metastatic tumours and in all the metastatic tumours, while E-cadherin was expressed in 17 out of 20 (85 %) of the non-metastatic tumours and in 8 out of 12 (66 %) of the metastatic tumours (Fig. 1a and b). The Ki-67 antigen was found to be expressed in tumour cell nuclei of 13 out of 20 (65 %) non-metastatic carcinomas. In case of metastatic tumours 11 out of 12 (91 %) cases were positive (Fig. 1c). The results of encoded expression of the analysed markers is presented in Table 1. Statistical analysis revealed that intense Ki-67 antigen expression was associated with intense MMP-2 expression in the non-metastatic tumors (*p* = 0.03) (Table 2).

**Table 1:** Expression intensities of studied markers grouped and encoded according to established assessment scales

	Number of cases (% of cases)					
Expression intensity	Non-metastatic MMP-2	Metastatic MMP-2	Non-metastatic E-cad	Metastatic E-cad	Non-metastatic Ki-67	Metastatic Ki-67
None	3 (15.0)	0 (0.0)	3 (15.0)	4 (33.3)	7 (35.0)	1 (8.4)
Weak	9 (45.0)	6 (50.0)	8 (40.0)	2 (16.7)	6 (30.0)	6 (50.0)
Moderate	5 (25.0)	5 (41.6)	2 (10.0)	3 (25.0)	6 (30.0)	3 (25.0)
Intense	3 (15.0)	1 (8.4)	7 (35.0)	3 (25.0)	1 (5.0)	2 (16.4)

**Table 2:** Distribution of studied antigens in relation to their intensity in metastatic and non-metastatic tumours (low = none + weak + moderate; high = intense)

Lesion and marker type	No.	Non-metastatic Ki-67		*p*-value	No.	Metastatic Ki-67		*p*-value
		High	Low			High	Low	
Non-metastatic MMP-2	20	No. (%)	No. (%)			No. (%)	No. (%)	
High	3	3 (100)	0 (0.0)	0.03				
Low	17	4 (23.5)	13 (76.5)					
Metastatic MMP-2	12				12			
High					1	1 (100)	0 (0.0)	0.41
Low					11	4 (36.4)	7 (73.6)	
Non-metastatic E-cadherin	20							
High	7	2 (28.5)	5 (71.5)	1.00				
Low	13	5 (38.4)	8 (61.6)					
Metastatic E-cadherin					12			
High					3	1 (33.3)	2 (66.7)	1.00
Low					9	4 (44.4)	5 (55.6)	

**Fig. 2:**
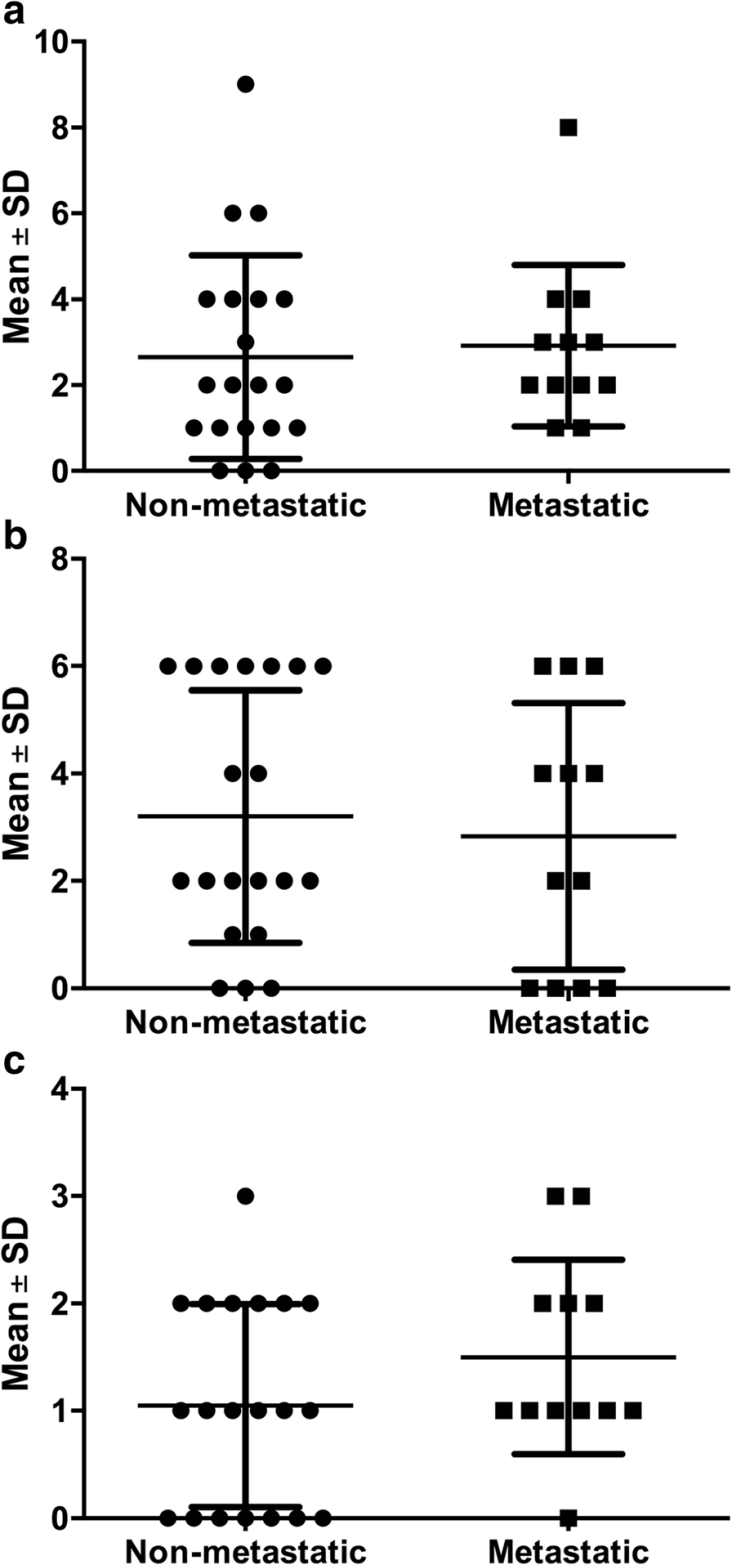
Immunoexpression of selected cell markers in non-metastatic and metastatic canine mammary carcinomas. Expression of MMP-2 (**a**), E-cadherin (**b**) and Ki-67 antigen (**c**), in non-metastatic and metastatic canine mammary carcinomas of female dogs. Data are presented as mean ± standard deviation (SD)

Higher expression of MMP-2 (2.9 ± 1.9 *vs* 2.7 ± 2 .4; *p* = 0.50; Fig. 2a) could be noted in the tumours that metastasized in comparison to that noted in the non-metastatic tumours. On the contrary, the expression of E-cadherin was lower in the tumours that metastasized (2.8 ± 2.5) compared to those that did not (3.2 ± 2.3). However, those differences were not statistically significant (*p* = 0.63; Fig. 2b). In accordance to the observed increase of Ki-67 antigen expression positivity with metastatic potential in the analysed tumours, its average expression was slightly higher in tumours that metastasized (1.5 ± 0.90 *vs* 1.1 ± 0.94; *p* = 0.22; Fig. 2c) than in those that did not. The Spearman’s correlations test did not show any statistically significant correlations between the expression intensities of the studied markers in the two groups of carcinomas.

Comparing metastatic and non-metastatic carcinomas, metastatic carcinomas showed lower E-cadherin expression and a high Ki-67 expression. This may indicate that the greater the number of mitotic neoplastic cells in the tumour, the weaker the intercellular connections (fewer E-cadherin adhesion molecules on their surface), and the easier it is for the cells to be released from the tumour. However, in our study, the observed trend did not reach statistical significance. Nevertheless, similar findings were demonstrated in a study of more than 100 women with breast cancer [14]. In that study, there was a complete loss of expression of E-cadherin in 64 % of lobular carcinomas and 19 % of ductal carcinomas. In our study, E-cadherin was not expressed in 34 % of metastasized and in 15 % of non-metastasized tumours. Interestingly we found that intense expression of MMP-2 was associated with intense Ki-67 antigen expression in the non-metastasizing tumours. Two explanations of this phenomenon are possible. First, these tumours may be regarded as being resected in direct preinvaseve state in which case the loss of E-cadherin expression could be the next step in acquirement of invasive properties. Secondly, this may be a random non-significant finding due to small sample size. Nevertheless, further analyses on larger cohorts are necessary to fully address this issue.

The expression of E-cadherin was found to be decreased in breast cancer and a positive correlation was found between its expression and the frequency of metastasis by Acs et al. and Bankfalvi et al. [24, 25]. Few studies were carried out on malignant breast tumours in dogs. They revealed that tumours with a decreased expression of E-cadherin grew more aggressively and metastasized more often [26, 27].

Our current findings indicate that the increase in the metastatic potential of tumour cells is associated with an increased expression of MMP-2 and a decreased expression of E-cadherin. These results are similar to our previous findings concerning the expression of MMP-9 [12]. However, the intensity of expression of those markers did not correlate. Nevertheless, our observations support the suggested biological role of these proteins in the metastatic process. The decrease in the expression of E-cadherin and increase in the expression of MMP-2 leads to a loss of cell cohesion and potentiates the degradation of the extracellular matrix. The lack of correlation between the expressions of the three proteins in our study was most likely due to the high biodiversity of canine mammary tumors. However, the role of E-cadherin, MMP-2 and Ki-67 in the metastatic process seems crucial.

The comparable expression of Ki-67 and MMP-2 that was obtained in our study in both metastasizing and non-metastasizing carcinomas may suggest an increased expression of MMP-2 in cells undergoing rapid division. Such co-expression may facilitate the disintegration of the stroma and the spread of rapidly dividing tumour cells. However, more detailed studies using an in vitro approach are warranted to elucidate this matter in canine mammary cancers.

That point of research was mostly addressed in human breast cancer, where the prognostic role of MMPs was intensively studied. Lu et al. [28] analysed 21 cases of mammary carcinomas in women and found that MMP-2 plays an important part in the metastasis of those tumours to regional lymph nodes. In addition, the authors found a significantly higher MMP-2 expression in the metastasis (lymph node) than in the primary tumour. An increased MMP-2 and MMP-9 expression was found in a number of tumours in humans. It is most often associated with unfavorable prognostic factors, such as the clinical advancement of the disease, an infiltration to the surrounding tissues, lymph node metastases and a shortened disease-free survival [29–35]. However, not all studies associate increased expressions of MMP-9 and MMP-2 in the tumour tissue with a poor prognosis. For example, there was a positive correlation between the presence of macrophages expressing high levels of MMP-9 in colorectal cancer and a decreased number of metastases [36]. Similarly, Zhang et al. [37] analysed close to 100 cases of invasive breast cancer in women and found no correlation between high expression of MMP-9 and adverse prognostic factors.

## Conclusion

We did not show significant differences in MMP-2, E-cadherin and Ki-67 expression between metastatic and non-metastatic tumours due to low number of cases studied, however further experiments are necessary to assess the role of these antigens in the process of canine mammary tumours metastasis. Furthermore, the obtained results may suggest that canine carcinomas may be used as an experimental model for future studies on the mechanism of carcinogenesis and metastasis in humans. Nevertheless, the results of this study should be regarded as preliminary, as the analysis was performed on a small number of cases.

## Abbreviations

BM, basement membrane; CAM, cellular adhesion molecules; E-cadherin, epithelial-cadherin; ECM, dense extracellular matrix; IL-1, interleukin-1; MMP-2, matrix metalloproteinase 2; N-cadherin, neuronal cadherin; P-cadherin, placental cadherin; PG, prostaglandin; RT, room temperature; TNF-α, tumour necrosis factor alpha; VEGF, vascular endothelial growth factor
